# Biofeedback for robotic gait rehabilitation

**DOI:** 10.1186/1743-0003-4-1

**Published:** 2007-01-23

**Authors:** Lars Lünenburger, Gery Colombo, Robert Riener

**Affiliations:** 1 Spinal Cord Injury Center, Balgrist University Hospital, Zurich, Switzerland; 2 Hocoma AG, Volketswil, Switzerland; 3 Rehabilitation Engineering Group, Swiss Federal Institute of Technology (ETH), Zurich, Switzerland

## Abstract

**Background:**

Development and increasing acceptance of rehabilitation robots as well as advances in technology allow new forms of therapy for patients with neurological disorders. Robot-assisted gait therapy can increase the training duration and the intensity for the patients while reducing the physical strain for the therapist.

Optimal training effects during gait therapy generally depend on appropriate feedback about performance. Compared to manual treadmill therapy, there is a loss of physical interaction between therapist and patient with robotic gait retraining. Thus, it is difficult for the therapist to assess the necessary feedback and instructions. The aim of this study was to define a biofeedback system for a gait training robot and test its usability in subjects without neurological disorders.

**Methods:**

To provide an overview of biofeedback and motivation methods applied in gait rehabilitation, previous publications and results from our own research are reviewed. A biofeedback method is presented showing how a rehabilitation robot can assess the patients' performance and deliver augmented feedback. For validation, three subjects without neurological disorders walked in a rehabilitation robot for treadmill training. Several training parameters, such as body weight support and treadmill speed, were varied to assess the robustness of the biofeedback calculation to confounding factors.

**Results:**

The biofeedback values correlated well with the different activity levels of the subjects. Changes in body weight support and treadmill velocity had a minor effect on the biofeedback values. The  synchronization of the robot and the treadmill affected the biofeedback  values describing the stance phase.

**Conclusion:**

Robot-aided assessment and feedback can extend and improve robot-aided training devices. The presented method estimates the patients' gait performance with the use of the robot's existing sensors, and displays the resulting biofeedback values to the patients and therapists. The therapists can adapt the therapy and give further instructions to the patients. The feedback might help the patients to adapt their movement patterns and to improve their motivation. While it is assumed that these novel methods also improve training efficacy, the proof will only be possible with future in-depth clinical studies.

## Background

### Robotic gait rehabilitation

Walking ability, though important for quality of life and participation in social and economic life, can be adversely affected by neurological disorders such as spinal cord injury, stroke or traumatic brain injury. Rehabilitation of patients with such disorders should include gait training because there is evidence that the desired function or movement has to be trained in a task-specific program [1,2]. One contemporary approach is body-weight supported treadmill training in which the patient is suspended over a treadmill and the patient's legs are guided by therapists [3-9]. Several studies have shown beneficial effects of this approach [10-12]. Because other studies [13,14] did not find an advantage compared to conventional therapy and systematic reviews [8,9] regard the evidence as controversial, further studies are required. There are some indications that an increased training intensity might lead to clearer results [15-18]. However, the manual form of this therapy in which the patient's legs are guided by two therapists holding and moving them along a gait-like trajectory is strenuous for the therapists and labor- and cost-intensive. Depending on the patient's condition, the therapists have to assist the stance leg by extending the knee against the weight of the patient or they have to flex the knee joint, possibly against spasticity, and lift the leg through swing phase. The high physical effort for the therapists often limits the training duration, whereas the patient might benefit from a longer duration. Recently developed rehabilitation robots [19,20] allow delivering continuous support for the legs in a physiological gait pattern, high repetition accuracy, and prolonged training duration compared to manual treadmill training. The loss of the physical contact between the therapist and the patient is a disadvantage, yet can partly be overcome by technology. The physical contact was often used by the therapist to "feel" the patient's ability and activity. With this information, the therapist can provide feedback to the patient, give training instructions and help to improve the patient's motivation. Because feedback on the current performance may improve the training effect [21], a corresponding, computerized feedback is desired for robotic rehabilitation. As *bio*logical quantities are transferred to a *bio*logical system (human) via artificial *feedback*, the term "biofeedback" has been introduced and became widely accepted.

The aim of this study was to develop a biofeedback system for a gait training robot and test its usability in subjects without neurological disorders.

## Feedback and motivation

### General considerations on feedback and motivation

To improve a certain motor function, it is helpful to know the level of your success and your performance. For human movements, this performance assessment is often derived from afferents and reafference such as proprioceptive, force or visual sensory inputs. They can also be described as intrinsic feedback [22]. This intrinsic feedback is generated by the movement itself (proprioception or vision of the moving limb, but also sound of the footsteps). In contrast, extrinsic or augmented feedback may be provided additionally by an outside source, such as a therapist or coach. This extrinsic feedback is important for learning some motor tasks [22]. For robotic rehabilitation, the robot itself can be used to generate and display the feedback.

Apart from its instructional aspect, feedback is also important for motivation. Keeping patients informed about their progress usually translates into greater effort during task practice [chapter 10 of ref. [22]]. This higher effort, e.g. in terms of enhanced endurance or higher compliance, might help to improve training outcomes. Pursuing and achieving goals usually motivates the subjects. This requires measurements to compare the current status with the desired goal. It is important to know the quantity and quality of the movements performed by the patient.

In neuro-rehabilitation, the neurological disorder can increase the need for artificial feedback. For people with neurological disorders, interpretation of intrinsic feedback could be difficult or incorrect due to impaired somatosensory pathways.

### Biofeedback principles in non-robotic gait rehabilitation

Biofeedback principles have been applied in gait rehabilitation of patients with stroke [23-31], cerebral palsy [32], spinal cord injury [33], Spina Bifida [34] or arthritis [35]. Electromyographic (EMG) recordings [23-26,32,33], kinematic quantities [25-30,34-38], and kinetic measures [37,38] have been processed and displayed visually [29,32], acoustically [27,28,30,37] or in combination [23,26,33,35,38], as well as via vibrotactile stimuli [34,36,37]. The application of biofeedback in stroke rehabilitation improved the patients' gait function according to a recent systematic review [8].

During manual training therapists can estimate the patients' performance in several ways. Apart from visual observation therapists can base this estimation on the amount of external assistance needed to perform the movement correctly. However, because the therapist will usually increase the assistance to maintain a physiological gait pattern when the patient's performance reduces, the patient does not have to walk with maximum effort (see also comments on motivation above). Conversely, many individuals with neurological disorders ambulate independently and might still benefit from training. For these individuals, assistance might be beneficial to achieve higher gait quality and delivers a basis for feedback. In conclusion, the estimation of (maximum) walking capability of the patient might be difficult with this assistance-based method. However, the estimation will reflect the current performance correctly. The feedback of this performance estimation might already be sufficient to enhance the training.

This approach based on required assistance can be translated to rehabilitation robots that are equipped with force sensors. However, the problems described above for the estimation by the therapist basically also apply to robotic implementation. With the most commonly used position-controlled strategies, these force sensors register the amount of robot-generated force assisting the patient to follow the predefined gait pattern. The use of these force or torque signals has an advantage over electromyographic muscle recording or standard videographic gait analysis, because no additional time or equipment is needed. Furthermore, electromyographic recordings register muscle activity. The movement resulting from this activity is usually difficult to identify especially when many muscles act onto the same joint and in dynamic situations like walking. Videographic gait analysis is limited by visual obstruction of the one leg by the other, or the rehabilitation device. Additionally, when position control strategies are applied, the visual gait analysis will mainly identify the underlying predefined trajectory. Therefore, we chose a force-based strategy described below for implementing a biofeedback for a gait rehabilitation robot.

## Force-based biofeedback in a rehabilitation robot

**Figure 1:**
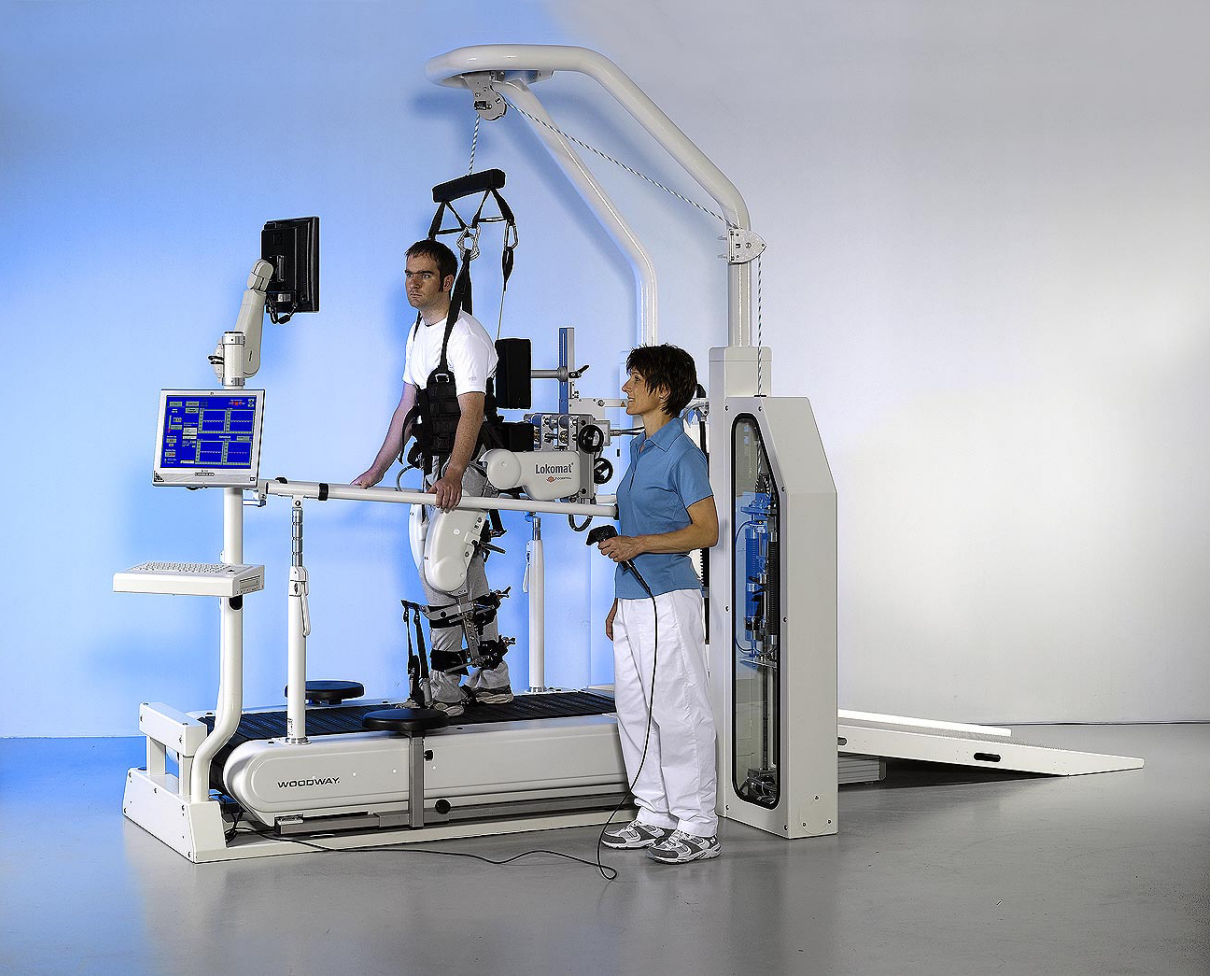
**The driven gait orthosis Lokomat**. The driven gait orthosis Lokomat Pro (Hocoma AG, Volketswil, Switzerland) is a bilateral robotic orthosis with actuated hip and knee joints that is used for body-weight supported treadmill training. (Photo courtesy of Hocoma AG, Volketswil, CH)

One specific strategy presented in this paper is based on a driven gait-orthosis DGO [20] (Lokomat^® ^Pro Version 4, by Hocoma AG, Volketswil, Switzerland). The DGO is a bilateral robotic orthosis that is used in conjunction with a body-weight support system to control the patient's leg movements in the sagittal plane (Fig. 1). The DGO's hip and knee joints are actuated by linear drives, which are integrated in an exoskeletal structure. A passive foot lifter induces an ankle dorsiflexion during the swing phase. The legs of the patient are moved with highly repeatable predefined hip and knee joint trajectories on the basis of an impedance control strategy [39]. Knee and hip joint torques of the patient are determined from force sensors integrated in the drives of the DGO.

### Implementation of the biofeedback

The technical implementation of a force-biofeedback strategy for the DGO has been described by the authors of this paper [39,40]. For this strategy, the subject's legs are guided by the DGO with high impedance (equivalent to position control). With this high stiffness, changes in the subject's behavior are best detectable because already small deviations lead to large counteracting torques by the robot. The torque outputs of the drives (with compensation for passive properties of the DGO) give direct information about the patient's activity and performance. If the patient actively moves according to the reference trajectory, no interaction torques from the subject would act on the robot. If the patient is passive and does not contribute to the walking movement due to paresis or lack of motivation, the robot has to exert torque in order to maintain the desired reference trajectory. Thus, the robot has to push the subject. Conversely, if the patient tries to move faster than the reference trajectory, the robot requires less torque or even has to decelerate the subject.

Biofeedback values are calculated for stance and swing phase of the gait cycle as weighted averages of the torques measured in the corresponding joint drives [39,40]. The appropriate selection of the weight functions leads to positive biofeedback values when the patient performs therapeutically desirable activities. Specifically, active hip flexion is required to bring the leg forward during the swing phase, active knee flexion during early swing phase and knee extension during late swing phase. During the stance phase, the most important activity is weight bearing by continuous, almost isometric knee extension, whereas hip extension results from a combination of muscle activity and passive motion of the treadmill. This means that for each joint, except the knee joint during stance phase, a torque pointing against the direction of movement should produce a negative feedback, one pointing parallel to the direction of motion a positive feedback. Mathematically this can be implemented by multiplication of the measured force and a weighting function for each time during the gait cycle. Integration of joint torques weighed according to this principle during phases of the gait cycle delivers values that are comprehensive in summarizing the performance in the specific gait phase and that are more robust against noise than the continuous signal. Similar scaling for all values is obtained by normalization (For the mathematical formula see [39]). Because weighting functions that are proportional to the angular velocity follow the described principle, the present implementation employs these functions for hip joint during stance phase and knee joint during swing phase, as well as hip joint during swing phase with a slight modification. This modification was implemented because there is some indication for a passive pendulum-like motion of the leg in mid swing [41]. It reduces the importance of this phase by multiplication of the weighting function with an additional smooth function (quenching). In contrast to these three biofeedback calculations, the weighting function for the knee during stance phase was chosen to be constant because it takes the requirement of constant weight bearing better into account. In summary, this biofeedback approach provides four biofeedback values per stride and per leg that become available immediately after each step.

**Figure 2:**
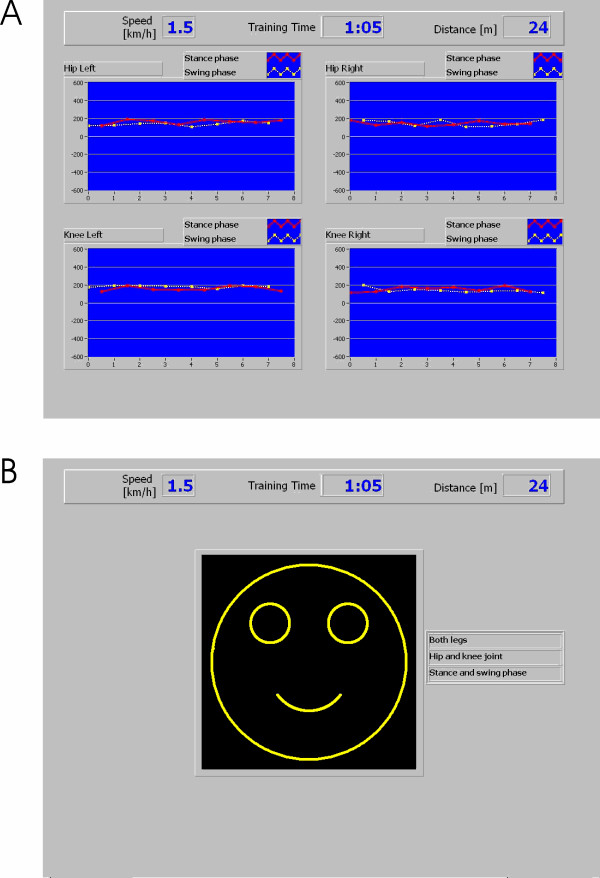
**Visual displays of the biofeedback**. Screen shots of two standard displays of the biofeedback implemented for gait training. Four biofeedback values become available after each step (e.g. left leg stance phase and right leg swing phase). These data can be displayed in a line diagram (A), which is updated twice per stride. Each point represents the biofeedback value of one stride. The values are displayed in independent subplots for each of the four joints. Swing and stance phase are color-coded. Both axes can be adjusted by the therapist in order to adapt the feedback to the current training situation. It is possible to display a selection of biofeedback values (e.g. only one leg, only swing phase, only knee joints) to help the patient focusing on specific aspects. The selected subset of biofeedback values can also be averaged into one value that can be displayed by a smiley (B) which smiles broader for higher and frowns for lower values of the biofeedback during the most recent step.

The most complete display shows all 8 values per stride in an array of line graphs (Fig. 2A), each including the history for a modifiable number of recent strides. This allows monitoring every aspect of gait performance that is evaluated by the biofeedback. For supervision, a similar visualization can be displayed on the therapist's monitor. Many patients understand quickly which movement leads to higher biofeedback values after verbal instruction by their therapists. However, recurrently reminding the patients usually improves their performance. Simultaneously, the visualization for the patient can be adapted to emphasize specific gait performance aspects and to avoid information overload for the patient. Specifically, the display should be accessible in the way that the patients are able to perceive the information displayed to them, i.e. large fonts readable while walking. The display should also be intuitive. Otherwise, additional time would be required for learning to understand and use the display and therefore shorten the available training time. Intuitive displays are even more important in neuro-rehabilitation because some patients with neurological disorders who require gait retraining also sustain cognitive deficits (e.g. after traumatic brain injury). Thus, such patients could benefit from a reduction to one value per gait phase and a visually more appealing display, such as a smiley face (Fig. 2B). The biofeedback values are summarized by averaging the values of a subset selected by the therapist. Averaging results in an overall factor that is relatively unbiased. In this way, the therapist can have the patient focus on specific aspects of walking. The possible performance loss in the remaining aspects of walking that are not selected for the feedback should be monitored by the therapists with the help of the complete display on their monitor. When selected, the smiley is continuously displayed on the monitor in front of the patient and updated every step. The shape of the smiley's mouth (an arc of a circle) is determined from the obtained average biofeedback value for the last step as well as threshold and scaling factors set by the therapist. For averages larger than the therapist's setting, the ends of the mouth point upward (smile), for averages below the threshold, the ends of the arc point downwards (frown). The arc lengthens with larger absolute values resulting in a more prominent smile or frown for high and low values respectively. The scaling factor allows the therapist to adjust the sensitivity of the feedback to the functional abilities of the patient. In conclusion, the smiley display allows for a goal-oriented training with feedback, i.e. the patient should focus on specific movements to reach the "goal" of a full smile.

### Validation in subjects without neurological disorders

Three subjects without neurological disorder (2 female, 1 male), aged 24–30 years, without neurological disorders were included in the study after giving informed consent and approval by the regional ethics committee of the Canton Zurich. The subjects walked in the DGO at two different speeds (1.8 and 2.4 km/h). A dynamic body-weight support system was used to support 25%, 50%, and 70% of subject's body weight. Apart from the optimal setting of the synchronization of the DGO and the treadmill, two other settings were used that caused the DGO either to walk about 10% slower or faster.

All subjects had previous experience in walking within the DGO. During recording times of 30 seconds, the subjects were instructed to walk in three different ways: (1) Passive: They should not contribute to the movement. (2) Active: They should walk with the same pattern as the DGO. (3) Exaggerated: They should exaggerate their movements in order to increase the biofeedback values that were displayed as line graphs. With the given time and endurance limitations, not all of the 54 possible combinations could be tested in the single session performed. Subject P1 completed 41, subject P2 45 and subject P3 42 trials. The actual joint angles and the joint moments were digitally recorded with a sampling rate of 1 kHz.

For analysis, biofeedback values were re-calculated offline (using Matlab, Mathworks Inc.) from the recorded torques according to the method described above, i.e. as weighted averages of the force values using the described weighting functions. (The analysis would have been possible by selecting strides from the automatically generated biofeedback file. The recalculation was done for convenience and easier automatic analysis). For illustration, the torques and angles were cut into strides and normalized in time to 100 samples per gait cycle. For purposes of correlation with recorded joint torques and biofeedback values using Spearman correlation in Matlab (Mathworks Inc.), the walking instructions were coded as "passive" = 0, "active" = 1, "exaggerated" = 2.

### Torques acting during walking in the robot

**Figure 3:**
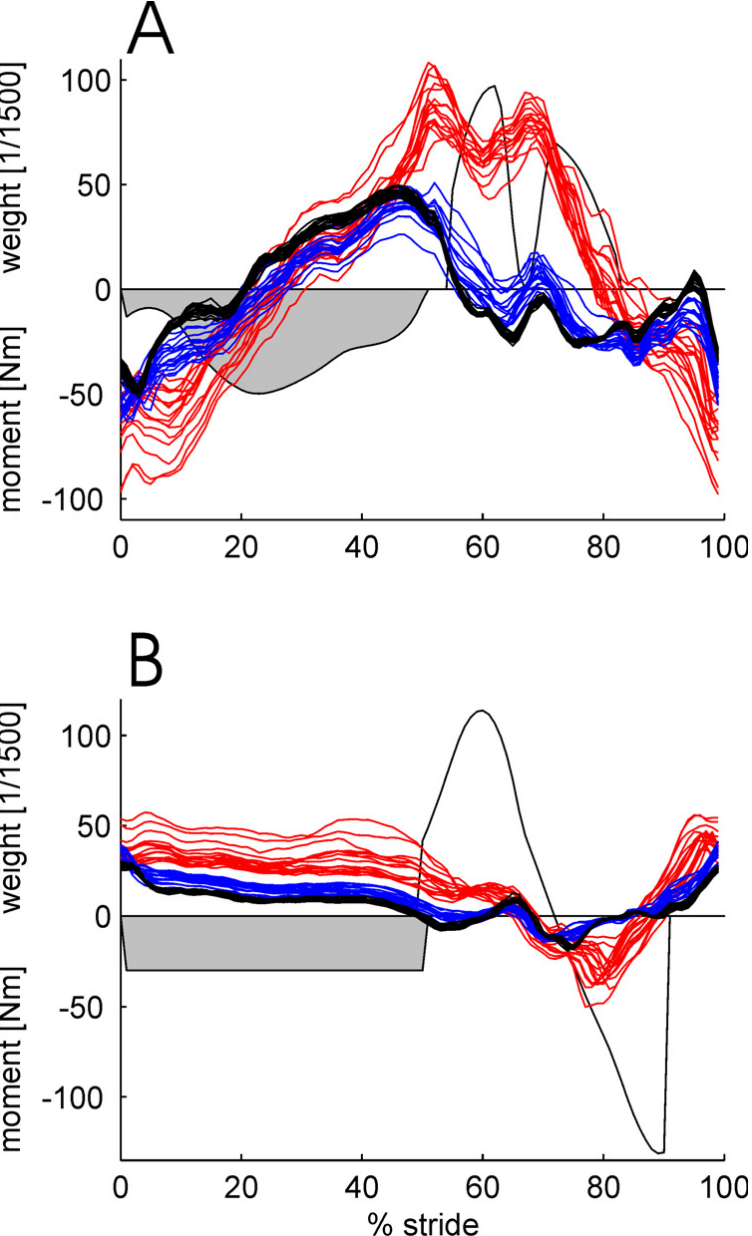
**Example traces of joint torques during walking in the robot with different instructions**. Joint moment in the hip and knee joint of the DGO were recorded while a subject without neurological disorders walked according to three different instructions The other parameters, treadmill speed, body weight support, synchronization between DGO and treadmill were held constant. The instructions were: Passive (black): Do not contribute to the movement. Active (blue): Walk with the same pattern as the DGO. Exaggerated (red): Exaggerate the movement pattern to increase the biofeedback values displayed to them as line graphs (red). The weight functions used for calculation of the biofeedback values are illustrated as shaded areas.

Torques in the DGO joints were recorded during walking with different instructed walking activity – passive, active, exaggerated – and different settings of body weight support, treadmill speed and synchronization coefficient of DGO and treadmill. The effect of different instructed walking activities on the recorded torques are shown for one example subject in Fig. 3. The traces show a large variability within the 11–12 steps in each condition. The largest variability was present in the "exaggerated" condition. The traces of the active condition are between the traces of the passive and those of the active conditions for most of the times.

**Figure 4:**
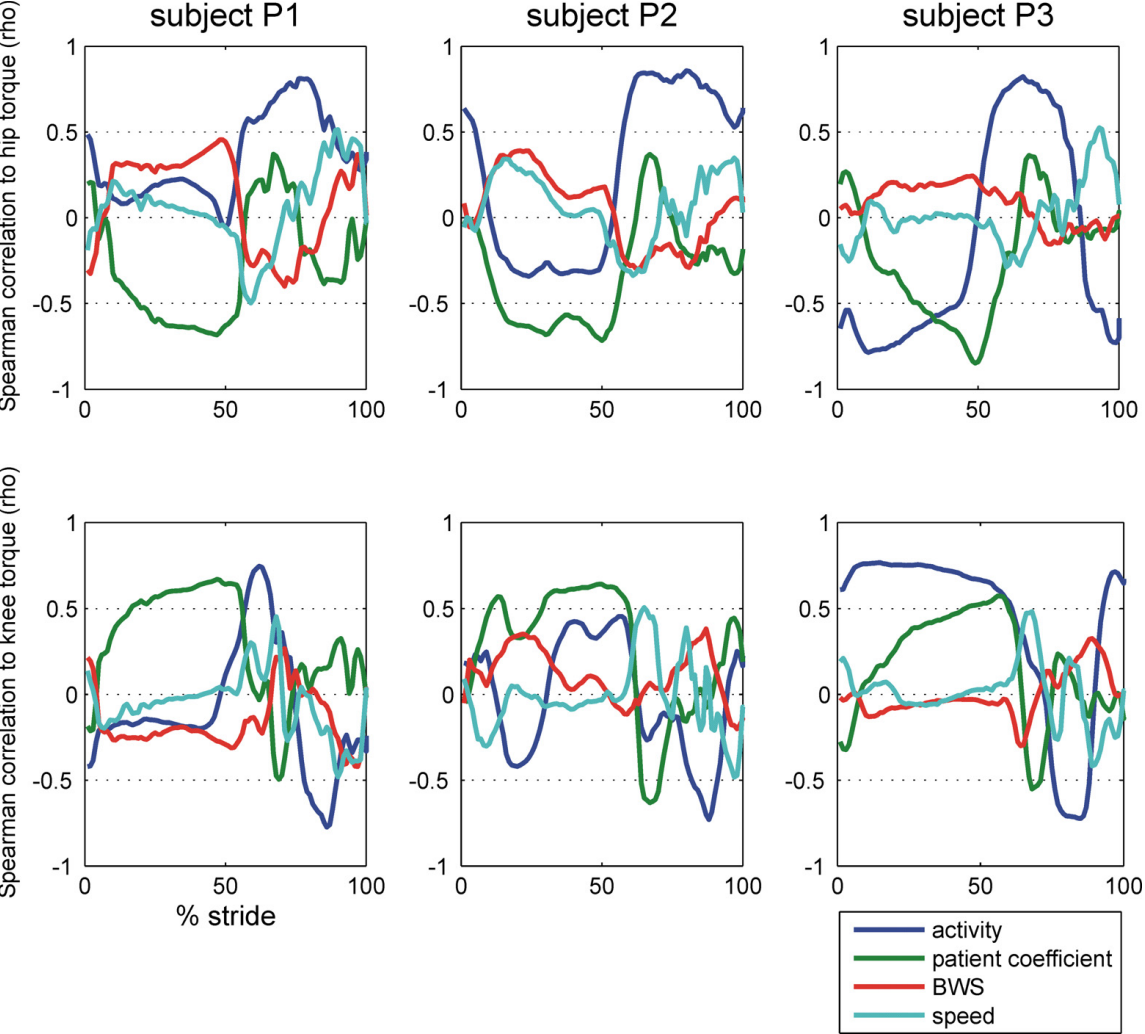
**Correlation of the joint torques with the walking parameters during the gait cycle**. The torques in the hip and knee joints of the DGO were recorded during the walking sessions of three subjects and correlated to the different walking instructions ("passive" = 0, "active" = 1, "exaggerated" = 2; blue) and different walking parameters: synchronization of robot and treadmill ("patient coefficient" optimal and +/- 5 units; green), body weight support (25%, 50%, 70% of body weight; red) and treadmill speed (1.8 and 2.4 km/h; cyan).

The correlation of the recorded torques at each time of the gait cycle and the four external parameters, instructed activity, patient coefficient, body weight support and treadmill speed were calculated and are shown in Fig. 4 for the right hip and knee of the three subjects. In all three subjects, the correlation of hip joint torque and instructed activity was high (>0.5) during swing phase ranging from about 55% to 100% of the gait cycle. The correlation of hip torque and activity was inconsistent during stance phase, being close to zero for 2 subjects and smaller than -0.5 for one subject. For the knee joint, the correlation of torque and activity was also small during stance phase. During swing phase, the correlation of knee torque and activity was positive during early swing, when the knee is flexing, and negative (<-0.5) during late swing when the knee is extending.

Changing the synchronization of DGO and treadmill influenced the hip and knee joint torques during the stance phase, especially at its end when the correlation coefficients were >0.5 for the hip and <-0.5 for the knee joint. The correlation coefficients of hip and knee torques and treadmill speed were generally close to zero during stance phase and had a consistent biphasic pattern during swing phase. The correlation coefficients of hip and knee torques and the amount of body weight support were generally closer to zero during the whole gait phase with largest values in the hip during the stance phase.

### Correlation of biofeedback and subject's activity

**Figure 5:**
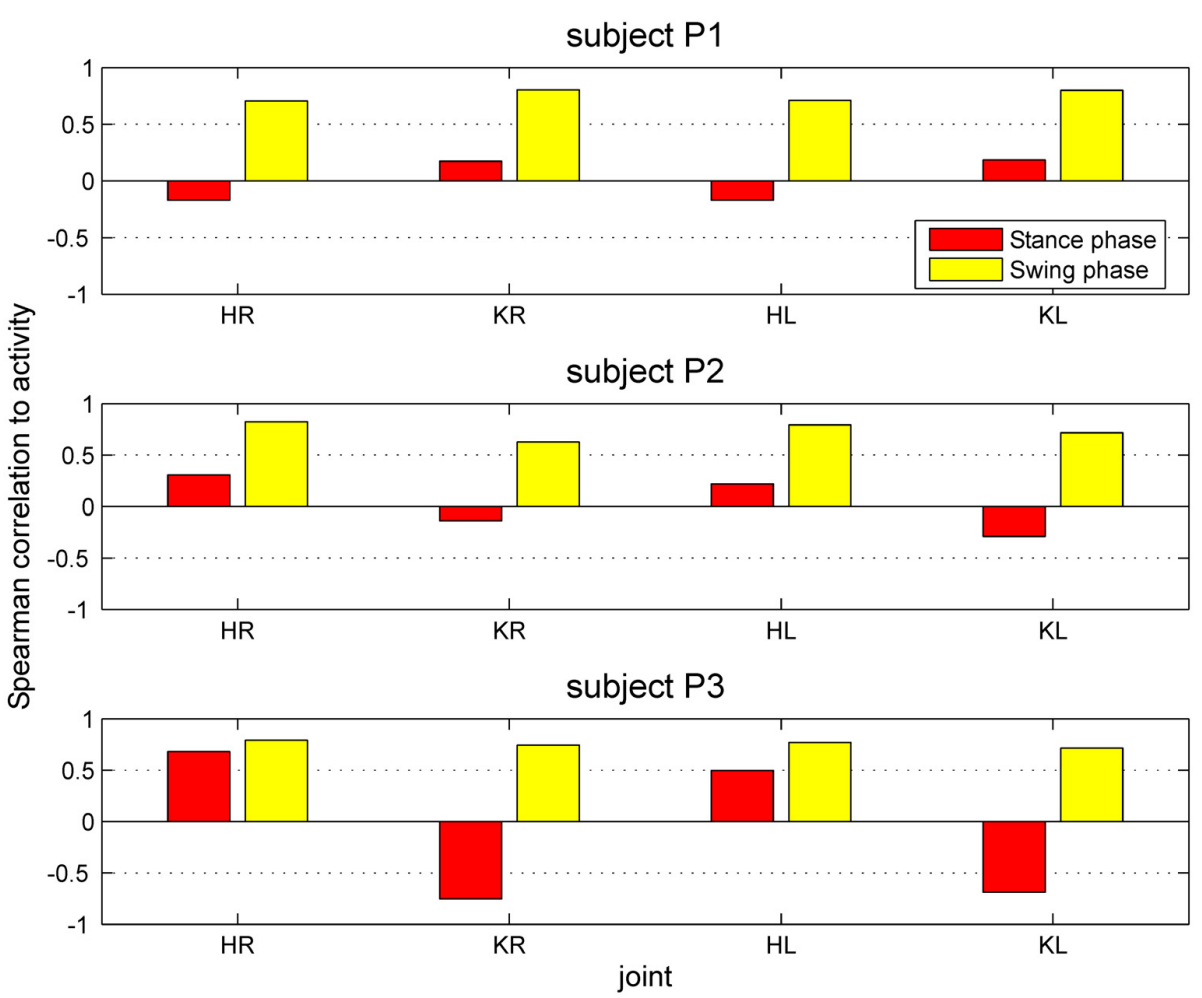
**Correlation of the biofeedback values with the instructed performance of subjects without neurological disorders**. Three subjects without neurological disorders were instructed to walk in the DGO with three different levels of activity (passive, active, exaggerated) and with different treadmill speed, body weight support and synchronization of DGO and treadmill. Spearman correlation coefficients of the biofeedback values obtained during this walking and the instructed activity are shown ("passive" = 0, "active" = 1, "exaggerated" = 2).

Biofeedback values were calculated as weighted averages using the weight functions described above and illustrated in Fig. 3. The resulting values for all four joints in two gait phases during about 580 strides for each subject were correlated to the level of activity the subject was instructed to perform (0 = passive, 1 = active, 2 = exaggerated). The reason to use the instructed level of activity was that no other quantification for gait performance was available that would allow a concurrent validation. The implied proposition that the subjects complied to the instruction is not a strong assumption. Spearman correlation coefficients were calculated because non-linear relations could be expected. The results are shown in Fig. 5 and Table 1. Biofeedback values of the swing-phase correlated highly with the instructed activity (range ρ = 0.63 to 0.82, mean ρ = 0.75; p < 0.01). The correlation of instructed activity and the biofeedback values of the stance-phase was lower (range ρ = -0.75 to 0.68, mean ρ = -0.01), especially in two subjects, and sometimes even negative. The negative correlation to the activity was not desired. However, it cannot be completely avoided with the present calculation method because the mechanical contact of the foot and the treadmill during the stance phase results in the passive torques acting onto the hip joint.

**Table 1:** Correlation of biofeedback and subject's activity

**Joint**	**Hip right**		**Knee right**		**Hip left**		**Knee left**	
**Gait phase**	**Stance**	**Swing**	**Stance**	**Swing**	**Stance**	**Swing**	**Stance**	**Swing**

Subject P1	-0.17	0.71	0.18	0.80	-0.17	0.71	0.19	0.80
Subject P2	0.31	0.82	-0.14	0.63	0.22	0.79	-0.29	0.72
Subject P3	0.68	0.79	-0.75	0.74	0.50	0.77	-0.69	0.72

Spearman correlation coefficients of the obtained biofeedback values and the level of activity with which they were instructed to walk in the DGO (0 = passive, 1 = active, 2 = exaggerated). In three individuals, correlations are show independently for the four actuated joints and for stance and swing phase.

### Other factors influencing the biofeedback

The correlation of biofeedback values and the synchronization settings of DGO and treadmill had large absolute values (max 0.68, mean 0.39), and were higher for the stance phase than for the swing phase. Because the synchronization of the leg movements and the treadmill influenced the forces between the treadmill and the stance leg, it also affected the joint torques. These torques are integrated into the biofeedback values, which indeed show a correlation to the synchronization setting.

The correlations of the biofeedback values to the amount of body weight support and to the treadmill speed are relatively small. For the body weight support, the absolute values of the correlation coefficients were on average 0.19 with a maximum of 0.38. For treadmill speed, the absolute values were on average 0.14 with a maximum 0.33.

The influence of gait parameters other than the subject's activity on the biofeedback values is therefore minor for values addressing the swing phase. The stance-phase values are strongly influenced by the synchronization of walking cadence and treadmill speed. The calculation of these values will be updated to improve the robustness against disturbances that is important for quantitative analysis. For the use as a biofeedback, however, this effect is less important because for adapting his or her motor activity the patient will concentrate on the last several steps and will take into account changes in the other parameters. Furthermore, the currently used weighting functions originate from basic biomechanical reasoning (as described above) and can be understood as a first-order approximation to robot-assisted walking.

### Clinical importance

Before trying to address the efficacy of the biofeedback for rehabilitation, it is useful to check the usability and the effect on compliance in patients. Preliminary results obtained from patients with SCI gave positive responses both from patients and therapists [39]. Six subjects with incomplete spinal cord injury walked with different instructions during five trials of 30 s each. They were instructed to walk as powerfully as possible in two trials. They were verbally instructed and motivated by a coach in one trial (no visual display), whereas they used the biofeedback display in the other trial (no verbal instruction and motivation). The biofeedback values during both active trials were significantly higher than during the passive control trials for 5 out of 6 subjects with only a little or no significant difference between the two active trials. One patient (the only one with ASIA impairment scale C [42]) was not able to substantially modulate the biofeedback and did not regain independent walking function during this therapy period. It was interpreted that the visual biofeedback is as effective as the continuous verbal instruction for the observed short time periods. Subjects reported in questionnaires that they felt positive about the biofeedback and wanted to use it again. However, it will be important to demonstrate clinical efficacy of the whole rehabilitation period and potentially faster rehabilitation with these new tools in future clinical studies.

### Extension to other technologies

Virtual reality techniques developing from visualization and simulation start to enter the rehabilitation domain [for review see [43]]. The techniques, including large screen 3D projections and head mounted display technology that allow depth perception, permit the immersion of the subject into an environment that is artificially generated in a computer. With an appropriate choice of the environment, it should be possible to instruct and motivate the subjects for training and rehabilitation. This enhanced motivation and feedback has the potential to improve the training efficacy and rehabilitation outcome.

## Conclusion

Biofeedback is a necessary addition to robotic gait training. It can provide an online feedback about the patients' performance to the training and allow the patient and the therapist to assess the walking performance. This can help to adapt and improve the training. The subjects might draw additional motivation from the online feedback on their performance.

Furthermore, the assessment of the patients' performance might be used not solely as online feedback, but also for evaluation of the rehabilitation progress. The integration of robot-aided training with robot-aided assessment and feedback has the potential to improve robotic rehabilitation.

## Abbreviations

DGO Driven gait orthosis

EMG Electromyography

## Competing interests

LL is employed by the University of Zurich via a CTI (Commission for Technology and Innovation) project funded by the Swiss Bureau of Education and Technology and Hocoma AG, Volketswil, Switzerland, which produces the Lokomat.

GC is founder, shareholder and CEO of Hocoma AG, Volketswil, Switzerland, which produces the Lokomat. GC is one of the inventors of the Lokomat.

## Authors' contributions

LL conceived and designed the study, recruited subjects, performed the acquisition and analysis of data, and drafted the manuscript. GC and RR provided expert guidance on experimental design, assisted with data interpretation, helped drafting the manuscript and edited the manuscript.
